# Stress-Induced Hyperglycemia Predicts Poor Outcomes in Primary Intracerebral Hemorrhage Patients

**DOI:** 10.3390/neurosci6010012

**Published:** 2025-02-02

**Authors:** Kevin Gilotra, Jade Basem, Melissa Janssen, Sujith Swarna, Racheed Mani, Benny Ren, Reza Dashti

**Affiliations:** 1 Renaissance School of Medicine at Stony Brook University, Stony Brook, NY 11794, USA; kevin.gilotra@stonybrookmedicine.edu (K.G.); jade.basem@stonybrookmedicine.edu (J.B.); sujith.swarna@stonybrookmedicine.edu (S.S.); 2 Department of Neurosurgery, Loma Linda University, Loma Linda, CA 92345, USA; mjanssen@llu.edu; 3 Department of Neurology, Renaissance School of Medicine at Stony Brook University, Stony Brook, NY 11794, USA; racheed.mani@stonybrookmedicine.edu; 4 Biostatistical Consulting Core, Renaissance School of Medicine at Stony Brook University, Stony Brook, NY 11794, USA; benny.ren@stonybrookmedicine.edu; 5 Department of Neurosurgery, Renaissance School of Medicine at Stony Brook University, Stony Brook, NY 11794, USA

**Keywords:** intracerebral hemorrhage, primary, outcome, hyperglycemia, stress, diabetes

## Abstract

Introduction: The current literature suggests hyperglycemia can predict poor outcomes in patients with primary intracerebral hemorrhage (ICH). Chronic hyperglycemia is seen in patients with pre-existing diabetes (DM); however, acute hyperglycemia in non-diabetic patients is defined as stress-induced hyperglycemia (SIH). This study explored the influence of hyperglycemia on outcomes of primary ICH patients both in the presence and absence of pre-existing DM. Methods: Data regarding admission glucose, pre-existing DM, inpatient mortality, and modified Rankin Scale (mRS) scores at discharge were available for 636 patients admitted to Stony Brook Hospital from January 2011 to December 2022 with a primary diagnosis of ICH. Regression models were used to compare outcomes between patients with admission hyperglycemia and/or pre-existing DM to a control group of normoglycemic and non-diabetic ICH patients. Results: Patients with SIH had higher inpatient mortality rates and worse mRS scores at discharge (*p* < 0.001). An association with higher mortality and worse mRS scores at discharge was also seen in patients with hyperglycemia secondary to DM, although the strength of this association was weaker when compared to patients with SIH. Conclusions: Our findings suggest that SIH may play a greater role in predicting poor outcomes at discharge rather than a history of poorly controlled DM with chronic hyperglycemia. To develop a more thorough understanding of this topic, prospective studies evaluating the effect of changes in serum glucose during hospital stay on short and long-term outcomes is needed.

## 1. Introduction

Primary intracerebral hemorrhage (ICH) is a devastating condition with a significant rate of mortality and disability [1]. ICH forms 10–15% of all strokes and often requires neurosurgical intervention in addition to comprehensive medical management, including strict blood pressure control, and the treatment of other underlying medical conditions [2,3]. The current literature has shown that a variety of metabolic conditions can contribute to worse outcomes in ICH patients [4]. Among them, diabetes mellitus (DM) and stress-induced hyperglycemia (SIH) are believed to be the most common metabolic derangements seen in ICH patients [5]. SIH is defined as an acute glycemic increase in response to significant injury for a patient without the diagnosis of DM [5,6]. In the current literature, a glucose level > 180 mg/dL in the setting of an acute stressor such as ICH is the typical cut-off for defining SIH in non-diabetic patients [7].

A number of observational studies have demonstrated that SIH is a predictor of mortality, hematoma expansion, and worse functional outcomes amongst primary ICH patients [8,9,10,11,12,13,14,15,16,17]. The results of INTERACT-2 demonstrated an independent association between both hyperglycemia and DM with 90-day disability and mortality [17]. This was further validated in two recent meta-analyses [18,19]. When accounting for the presence or absence of DM, the majority of studies have shown that SIH predisposes to worse outcomes among ICH patients [14], although others found hyperglycemia in diabetic patients also follows this trend when compared with normoglycemic diabetic patients [9,20]. Nevertheless, numerous studies to date have found contradictory outcomes regarding the significance of admission SIH, its long-term predictive potential, and its effect when compared to a diabetic population [21,22,23,24,25,26].

In the present study, our goal was to further explore the association between acute and chronic elevated glucose states with early outcomes in primary ICH. We analyzed patients with primary ICH by categorizing them into different groups based on the presence or absence of previous DM diagnosis and SIH, as defined by admission blood glucose level. We hypothesize that the presence of SIH on admission will be associated with increased disability and mortality.

## 2. Materials and Methods

Medical reports and imaging data were collected retrospectively for all adult patients admitted to Stony Brook University Hospital that presented with intracerebral hemorrhage from January 2011 to December 2022. Patient privacy was ensured by identifying each patient in our database with a unique coded number. Inclusion criteria were (1) diagnosis of primary ICH and (2) age over 18 years old. Exclusion criteria were (1) ICH secondary to underlying vascular lesions, tumors, or trauma, (2) hemorrhagic conversion of ischemic stroke, (3) missing diagnosis of DM on admission, (4) missing blood glucose levels on admission, and (5) withdrawal of care or pronouncement of death on admission.

Collected data included basic characteristics, demographics, past medical history, pre-existing medical conditions, laboratory values on arrival and during admission, radiographic findings, and hematoma locations and volume. The diagnosis of DM for each patient was determined based on the presence or absence of an ICD code for DM in each patient’s chart or if DM was discussed when the patient’s initial history was taken upon presenting to the hospital. It is important to note that the prevalence of undiagnosed DM in the population is roughly 1–2% [27]. Modified Rankin Scale (mRS) and intracerebral hemorrhage (ICH) scores on admission and discharge were collected as well as social factors such as Do Not Resuscitate/Intubate (DNR/DNI) status and location of discharge [28,29].

**Figure 1:**
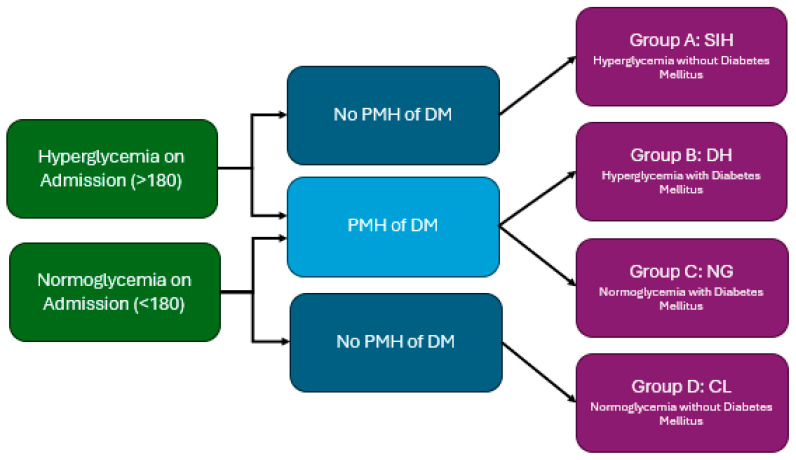
Groups A through D for study design with presence or absence of hyperglycemia on admission and DM indicated. SIH—stress-induced hyperglycemia, DH—diabetic hyperglycemia, NG—normoglycemia, and CL—control.

Patients were categorized into four groups based on previous diagnosis of DM and the presence of admission hyperglycemia. SIH was defined as admission glucose >180 mg/dL in non-diabetic patients, which is in line with the previous literature that has used cut-offs ranging from 140 to 200 for diagnostic purposes [7]. Since there is no universal agreed upon cut-off for SIH, 180 mg/dL served as a middle-ground cut-off based on the various definitions used in the literature [7]. Figure 1 highlights the categorization of patients. The four groups were defined as Group A—(SIH): blood glucose levels on admission above 180 mg/dL without history of DM, Group B—diabetic hyperglycemia (DH): blood glucose levels on admission above 180 mg/dL on admission with previous history of DM, Group C (DM only)—normoglycemia on admission with a history of DM, and Group D (control)—normoglycemia on admission without history of DM. As previously described in the CHEERY study with a similar methodology [14], the purpose of organizing the groups this way was to determine whether poor outcomes amongst diabetic patients occurred independently of SIH and, similarly, if poor outcomes amongst patients with admission hyperglycemia occurred due to SIH or hyperglycemia from underlying DM. For Groups A and B, we homogenized our cut-off for both SIH and admission hyperglycemia at a cut-off of 180 mg/dL to compare diabetic patients and non-diabetic patients more effectively with hyperglycemia on admission.

The two primary endpoints were inpatient mortality and level of disability at discharge, which was defined by two groups of mRS 0–2 and mRS 3–6 at discharge, with each being considered as a “favorable” or “unfavorable” outcome, respectively [13].

For the ordinal regression analysis, Groups A, B, and C were compared to reference Group D as these patients had neither SIH, admission hyperglycemia, or DM. We controlled for age, sex, and ethnicity for each regression analysis. We used the statistical software R (version 4.3.2) for our analysis of dichotomous and ordinal outcomes. Predictor variables were tested for multicollinearity in the logistic regression model using R software. Ordinal logistic regression and standard logistic regression allow us to compare odds ratios for group effects on the same scale [30,31,32]. This form of analysis has been well established throughout ischemic and hemorrhagic stroke literature for evaluating outcomes between patients with specific characteristics [30,31,32]. Our model was validated both internally and externally to prevent overfitting. Five-fold cross validation was used in a similar fashion to other studies in the stroke literature [33]. The proportional assumption was reached.

## 3. Results

In total, 814 consecutive patients presented with ICH during this period. Based on exclusion criteria, 636 patients with primary ICH and available glucose levels were included in this retrospective analysis. Table 1 highlights the characteristics of the four groups of patients in this study. Patients were stratified into one of four groups (Figure 1): non-diabetic patients with SIH (Group A, N = 42), diabetic patients with hyperglycemia (Group B, N = 70), diabetic patients without hyperglycemia (Group C, N = 101), and non-diabetic patients without SIH (Group D, N = 423). The inpatient mortality rate amongst the entire sample was 16.6%. A total of 6.6% patients had SIH (mean age = 74, male/female = 25/17), of which 45.2% expired in hospital. A total of 17.6% patients had hyperglycemia, of which 36.6% expired in hospital. A total of 22.9% patients had DM, of which 21.1% expired in hospital. When looking within each group, patients with SIH/hyperglycemia with and without DM had nearly double the percentage of mortality compared to the corresponding groups without SIH/hyperglycemia. Group A patients were primarily male (59.5%), while the majority of Group B and Group C patients were female (57.1% and 58.4%, respectively).

**Figure 2:**
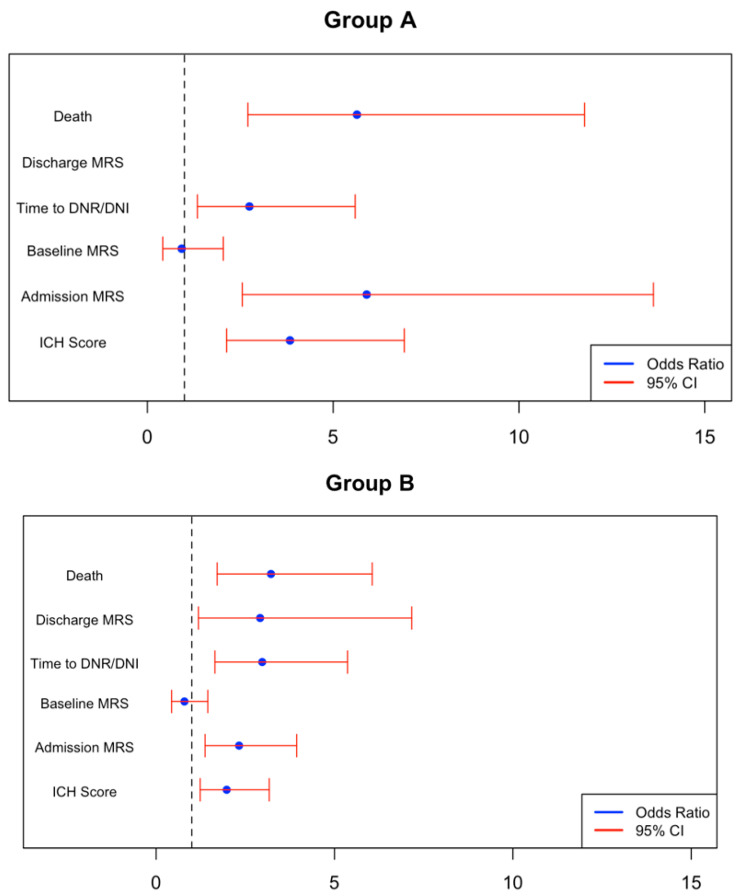

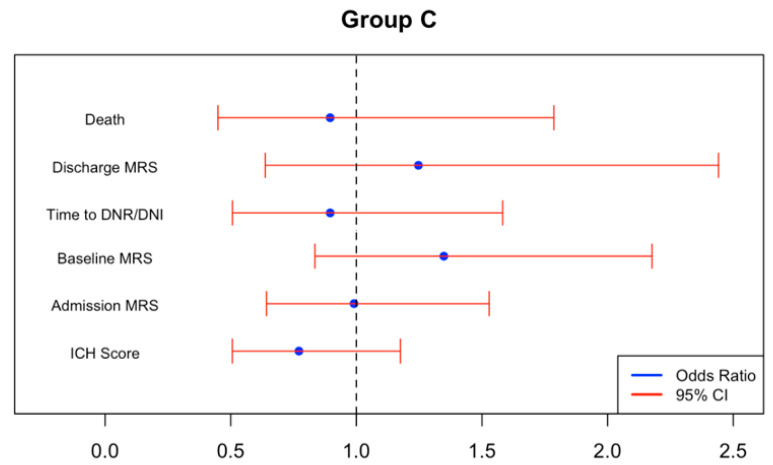
(**Groups A**–**C**): Multivariate ordinal regression analysis with odds ratios (OR) and 95% CI for **Group A** patients (SIH), **Group B** patients (DH), and **Group C** patients (NG). OR bounds can be calculated by exponentiating the 0.95 bounds from Table 2. All regression models adjusted for history of HTN, HLD, CAD, CHF, obesity, prior ICH, AF, malignancy, anticoagulation, and antiplatelet therapy. Odds ratio, OR = 1, is plotted as the vertical dashed line with any OR > 1 being considered as an unfavorable outcome. Plot scales were limited to aid visualization of the plots.

We generated logistic and ordinal logistic regression models that controlled for a variety of pre-existing comorbidities based on what was deemed important in prior studies [14,30,31,32], age, sex, and ethnicity. Ordinal outcomes were reordered to maintain the same direction of effect for adverse outcomes as standard logistic regression. In our analysis, positive coefficients correspond with an increased probability of adverse clinical outcomes. These included hypertension (HTN), hyperlipidemia (HLD), coronary artery disease (CAD), congestive heart failure (CHF), obesity, prior primary ICH, atrial fibrillation (AF), hematoma location (B and C), malignancy, and the presence of anticoagulation or antiplatelet therapy. Figure 2 highlights the ordinal regression coefficients for Groups A–C to assess their correlation with the primary outcomes (excluding patients without DM and without SIH).

A statistically significant association was seen between inpatient mortality and both Group A (*p* < 0.0001) and Group B patients (*p* = 0.0003) but not in the other two groups. Discharge to hospice, acute care facilities, and rehabilitation centers was observed significantly more amongst both Group A (*p* = 0.011) and Group B patients (*p* = 0.0016). There was no association between initial hematoma volume and admission glucose or a history of DM seen across the four groups. When comparing Group A and B, the odds ratios for Group A were higher than what was seen for Group B regarding all primary outcomes, including inpatient mortality and unfavorable discharge mRS. Group A was noted to have higher mRS scores on admission (*p* < 0.0001), but no association was found with hematoma volume or ICH scores. The remainder of the groups demonstrated no statistically significant association with either hematoma volumes, ICH scores, or mRS scores for their presentation on admission. Notably, ORs were also higher for Group A when assessing the severity of presentation in comparison to Group B, as demonstrated by worse mRS scores and ICH scores at admission.

## 4. Discussion

Admission hyperglycemia and SIH are both associated with worse outcomes in primary ICH patients [17,19]. Furthermore, SIH is thought to be a compensatory response to an acute stressor, such as ICH, in patients without a previous diagnosis of diabetes [6]. It has been hypothesized that the catecholamine surge in conjunction with many other hormones, particularly cortisol, is responsible for mediating this process by inhibiting glucose uptake into skeletal muscle and increasing gluconeogenesis in the liver [6]. Although the purpose of this acute adaptation is to provide the body with more energy in the setting of a physical stressor, acute hyperglycemia can simultaneously cause significant damage to neuronal tissue through oxidative stress and inflammation [6]. Other proposed mechanisms for how hyperglycemia leads to neuronal damage include the inhibition of cellular proteins such as aquaporin-4 and kallikrein [34,35]. Neuronal cell damage can proceed in a similar fashion with diabetes-induced inflammation, which is also largely mediated by oxidative stress [34,35]. 

The European Stroke Organization recommends controlling serum glucose through intravenous insulin infusion in a manner that mimics normal physiology while avoiding boluses with high-dose subcutaneous insulin [36]. Despite this recommendation, however, some studies have found that tight glycemic control does not necessarily lead to a major improvement in either short-term or long-term outcomes [37,38]. In an RCT of 78 patients with SAH, intensive insulin therapy did not lead to significantly improved outcomes [37]. A larger meta-analysis of 1248 neurocritical care patients further demonstrated that intensive insulin therapy raises the risk of hypoglycemia and offers minimal mortality benefit [38]. Glycemic control in the inpatient setting is often performed on a case-by-case basis where the patient’s pre-existing medical history, baseline glucose levels, and overall clinical status are collectively taken into consideration. The lack of consensus on how glucose should be monitored in these patients may be attributed to few studies comparing outcomes amongst non-diabetic and diabetic ICH patients with SIH/hyperglycemia.

To address this management issue, the question now becomes whether poor outcomes in ICH patients are due to the deleterious effects of pre-existing chronic hyperglycemia as seen in poorly controlled diabetics or because of the inflammation, edema, and hematoma expansion that occurs in the setting of SIH. While both factors are likely to play a role in these outcomes, the current literature pertaining to this topic is both limited and somewhat conflicting. Godoy et al.’s study of 510 ICH patients demonstrated no significant differences in mortality when comparing those with admission glucose > 160 mg/dL to those with admission glucose < 160 mg/dL [23]. On the contrary, Kimura et al. demonstrated that an admission glucose > 150 mg/dL independently predicted inpatient mortality in a sample of 100 ICH patients [8]. Meanwhile, another study highlighted that hyperglycemia that persists throughout a hospital stay is associated with higher mortality rates when compared to transient hyperglycemia or normoglycemia [24]. The INTERACT-2 study showed that both hyperglycemia and DM were independently associated with long-term disability at 90 day follow-up and higher mortality rates [17].

Few studies have comprehensively compared outcomes between ICH patients with normoglycemia to ICH patients with elevated glucose levels due to SIH or hyperglycemia in the setting of DM. Results from this study showed that patients with SIH (Group A) had a significantly higher risk of unfavorable outcomes when compared to normoglycemic and non-diabetic patients (Group D). Regression coefficients from Table 2 show that Group A patients had a stronger association with inpatient mortality and unfavorable discharge status compared to all other groups. Both Group A and Group B patients with admission hyperglycemia and DM were significantly more likely to present with higher mRS scores and ICH scores on admission, indicating more severe presentations. Unlike previous studies, which set arbitrary cut-offs of hyperglycemia ranging from 140 to 200 mg/dL, we used the same cut-off of 180 mg/dL to define hyperglycemia in non-diabetic patients, which was deemed as SIH (Group A) and diabetic patients (Group B). Even after homogenizing the cut-off value, our results yielded regression coefficients that demonstrated a much stronger association with unfavorable outcomes amongst ICH patients with SIH (Group A) when compared to regression coefficients for ICH patients with DM and admission hyperglycemia (Group B). In other words, while the combination of DM and admission hyperglycemia was associated with worse outcomes amongst Group B patients, the strength of this association was weaker when compared to the presence of SIH alone in Group A patients. This may support the notion that SIH has a more prominent effect on poor outcomes in ICH patients than hyperglycemia due to DM. Importantly, our study’s findings are very similar to Sun et al.’s study, which demonstrated that admission glucose was predictive of poor outcomes at 3 months of follow-up in non-diabetic patients, but admission glucose was not quite predictive in diabetic patients [20].

While poor outcomes associated with SIH may be due to rapid maladaptive pathophysiological changes, chronic hyperglycemia in the setting of DM can also affect outcomes through a more insidious process. Atherosclerotic changes to cerebral microvasculature and endothelial damage can predispose to vessel rupture and subsequently higher volumes of bleeding [14]. Perihematomal cell necrosis, cerebral edema, and concomitant hematoma expansion in diabetic ICH patients may all serve as plausible mechanisms for why chronic hyperglycemia is associated with poor outcomes [39,40]. SIH, on the other hand, causes deleterious effects through stress and inflammatory responses that promote the formation of microthrombi that worsen reperfusion injury. At the cellular level, this is associated with the accumulation of lactate and dysfunctional mitochondria, and all these findings collectively may explain the adverse outcomes seen in ICH patients with SIH [41]. Additionally, poor outpatient glycemic control may further propagate worse outcomes in diabetic patients who already have a degree of vascular stiffness that is worse than patients presenting in acute hyperglycemic crises [42]. Pre-existing vascular stiffness may explain why patients with DM are at higher risk of hematoma expansion and are pre-disposed to larger hematoma volumes. 

Despite this evidence, our study’s results showed no significant associations between unfavorable outcomes amongst patients with a history of DM who had normoglycemia on admission (Group C). This trend may have occurred because Group C patients had relatively well-controlled diabetes, as demonstrated by their normoglycemia on admission. It is important to note that in many prior studies, ICH patients with DM are often compared to those without DM, with little consideration given to serum glucose levels [9,12]. By comprehensively evaluating serum glucose levels through four individual strata in our study, our results may further support the notion that the deleterious pathophysiology of SIH has a greater effect on outcomes amongst ICH patients than the chronic vasculopathy that occurs secondary to DM. From a disease management perspective, this further emphasizes the importance of providers aggressively controlling serum glucose among ICH patients as poor glycemic control could predispose one to worse outcomes. Whether this entails the early implementation of insulin or oral antihyperglycemic agents will require further prospective studies to see which forms of medical management lead to the best outcomes.

Notable strengths of this study include a relatively large sample size amongst each of the individual four strata. This allowed us to perform a complex regression model that compared four groups in an analysis that has rarely been performed in the current literature [14]. In our regression analysis, we controlled for the presence of multiple CVD risk factors, which may have served as confounders of poor outcomes in ICH patients in previous studies.

### Limitations

Our study has a few limitations. Firstly, the retrospective nature of the study limits our ability to determine a direct cause-and-effect relationship between hyperglycemia and poor outcomes.

Additionally, this was a single-center analysis, and our population mainly consisted of Caucasians. Our results should be interpreted with caution in regard to institution-dependent bias. Furthermore, this manuscript did not assess long-term follow-up.

Another limitation was that pre-admission blood glucose levels and baseline HBA1C values were not available, thus making it difficult to discern the patient’s baseline glycemic control and potential chronic hyperglycemia prior to admission. Without pre-admission glucose, an elevated admission glucose in the absence of DM may have been classified as SIH, even if the lab value was in line with the patient’s baseline glucose in an outpatient setting. Serum cortisol, another acute stress marker, was also not recorded, although this may serve as a valuable metric to evaluate in future prospective studies along with HbA1C. Specifically, it is integral to determine whether adverse outcomes in ICH patients are more common when HbA1C and serum cortisol on admission exceed a certain threshold. This would provide great insight into how hyperglycemia should be managed for ICH patients in both the inpatient and outpatient settings. We also could not obtain the duration of pre-existing DM in patient-years or distinguish between type one or type two DM.

Finally, regarding our statistical analysis, because ordinal regression is widely used in the literature [43], we leave further extensions beyond standard ordinal regression to future work.

## 5. Conclusions

The results of this study demonstrate that SIH may be associated with poor outcomes in ICH patients regardless of their history of DM, although patients without DM had a stronger association with poor outcomes. Glycemic control for non-diabetic patients with SIH greatly differs from those with DM and pre-existing chronic hyperglycemia. SIH patients are given insulin boluses to keep their glucose levels below 180 mg/dL, while diabetic patients need additional insulin on top of their basal–bolus regimen and regular diabetic medications. To develop a thorough understanding of the effects of DM and SIH in the role of ICH, larger randomized controlled trials evaluating changes in serum glucose during hospital stay in response to different forms of medical management, and then evaluating long-term outcomes, are needed.

## Figures and Tables

**Table 1:** Demographic characteristics and clinical outcomes for patients in all four groups.

Demographic Information (N = 636)	Group A—Hyperglycemia Without DM (SIH) (N = 42)	Group B—Hyperglycemia with DM (N = 70)	Group C—Normoglycemia with DM (N = 101)	Group D—Normoglycemia Without DM (N = 423)
Median age	74	71	75	74
Sex				
Female	17 (40.5%)	40 (57.1%)	59 (58.4%)	214 (50.6%)
Male	25 (59.5%)	30 (42.9%)	42 (41.6%)	209 (49.4%)
Race/Ethnicity				
African American	0 (0%)	8 (11.4%)	10 (9.9%)	33 (7.8%)
Asian	0 (0%)	1 (1.4%)	7 (6.9%)	6 (1.4%)
Hispanic	0 (0%)	3 (4.3%)	3 (3.0%)	7 (1.7%)
White	42 (100%)	58 (82.9%)	81 (80.2%)	377 (89.1%)
Clinical Outcomes (N = 636)				
Inpatient mortality	19 (45.2%)	22 (31.4%)	14 (13.9%)	51 (12.1%)
mRS discharge 0–2	0 (0.0%)	7 (10.0%)	20 (19.8%)	98 (23.2%)
mRS discharge 3−6	42 (100%)	63 (90.0%)	81 (80.2%)	323 (76.4%)

**Table 2:** Multivariate logistic and ordinal logistic regression coefficients for Groups A–C (Group D excluded as healthy reference group). Regression coefficients, 95% CI, and *p*-values were included after controlling for covariates. Group A was not included in the regression due to complete separation, where Group A only consisted of cases in Table 1.

Outcome	Group A—Hyperglycemia Without DM (SIH) (95% CI)	*p*-Value	Group B—Hyperglycemia with DM (95% CI)	*p*-Value	Group C—Normoglycemia with DM (95% CI)	*p*-Value
Inpatient mortality	1.730 (0.995, 2.465)	<0.001	1.169 (0.537, 1.801)	<0.001	−0.110 (−0.801, 0.580)	0.754
Unfavorable mRS discharge (3–6)	16.338 (−1076.4–1109.1)	0.976	1.070 (0.171, 1.969)	0.020	0.221 (−0.451, 0.893)	0.520
Baseline mRS	−0.079 (−0.872, 0.714)	0.845	−0.231 (−0.833, 0.372)	0.453	0.299 (−0.180, 0.778)	0.222
Admission mRS	1.775 (0.939, 2.611)	<0.001	0.844 (0.317, 1.371)	0.002	−0.009 (−0.443, 0.424)	0.966
ICH score	1.345 (0.756, 1.934)	<0.001	0.682 (0.211, 1.154)	0.005	−0.260 (−0.680, 0.161)	0.227

## Data Availability

The original contributions presented in this study are included in the article. Further inquiries can be directed to the corresponding author.

## Abbreviations

ICH—intracerebral hemorrhage, SIH—stress-induced hyperglycemia, DM—diabetes mellitus, mRS—modified Rankin Scale, HTN—hypertension, HLD—hyperlipidemia, CAD—coronary artery disease, CHF—congestive heart failure, AF—atrial fibrillation
